# A Course of Clinical Lectures Delivered at the Hotel Dieu, Paris, for the Session 1842-’3

**Published:** 1843-11-25

**Authors:** A. F. Chomel

**Affiliations:** Paris


### THE MEDICAL EXAMINER,

### And Retrospect of the Medical Sciences.

Vol. VI.] PHILADELPHIA, SATURDAY, NOVEMBER 25, 1843. [No. 23.

### A COURSE OF CLINICAL LECTURES,

*Delivered at the Hôtel Dieu, Paris, for the Session* 1842–'43.

BY A. F. CHOMEL, M. D.

LECTURE XIV.

RESUMÉ OF THE ANNUAL CLINIC.

(Concluded.)

DISEASES OF THE HEART.

The affections of the heart have been very numerous this year; there have been forty-one cases, out of which sixteen died. Out of these forty-one cases sixteen were men, and twenty-four women. This proportion of women, even taking into consideration the greater number of beds in the women's wards, is so great as to indicate some particular cause exercising its influence over the women. It is probable that this influence may be partially referred to their extreme sensibility, and to the acute and powerful manner in which they are affected by moral emotions. In eleven cases we observed symptoms of simple hypertrophy, six out of these eleven died. Autopsy in these six cases, revealed in some, an insufficiency of the valves, in the others there was no appreciable lesion of the heart. In sixteen cases during life we were able to detect symptoms of insufficiency of the valves; in four cases we ascertained symptoms of left auriculo-ventricular contraction, and in three cases a contraction and insufficiency of auriculo-ventricular.

Out of the total number of these affections twenty-nine times the lesions were observed on the left side. These results are confirmed daily by necroscopical observations, which prove that the material lesions are much more frequently found in the left side of the heart than in the right. Five patients died with all the symptoms of insufficiency of the valves. We had observed during their life time at the first sound of the heart, a blowing sound, which lasted for some moments. It is difficult in autopsies to detect insufficiency of the mitral valve. We can discover with facility the insufficiency of the sigmoid valves, by means of a jet of water poured into the aortic orifice, and which, in mechanically distending the valves, enables us to detect their insufficiency, but this experiment cannot be made in the case of the mitral valve.

In two cases of auriculo-ventricular contraction, a blowing sound could be heard commencing with the first sound of the heart, and finishing after it. Autopsy confirmed our diagnosis. In two other cases of auriculo-ventricular contraction, together with insufficiency, the blowing sound commenced before the first sound of the heart, and finished with the second sound. Finally, in one case where we heard the blowing sound at the first sound of the heart, near its apex, we diagnosticated an insufficiency of the sigmoid valves; but this diagnosis was not confirmed by autopsy, which seemed contrary to all known results. In six cases there were anormal sounds and irregularity in the pulse. When this irregularity in the pulse continues, it appears to us to indicate that there is an alteration in the valves of the heart or that it is about taking place. We have already referred to the relation which may exist between the lesions of the heart and rheumatic affections, we will only add a few words to what has been said on this subject.

Out of thirty-four cases of articular rheumatism, there have been fourteen complicated with an anormal condition of the heart. These complications of a morbid state of the heart, terminate often very happily; nevertheless, we generally hear a grating sound more or less sharp. This anormal sound does not then always indicate an organic lesion of the heart, but merely sometimes a transient morbid state, probably owing to a nervous disturbance of this organ. The observations which have been relied upon by those who have established a theory on this coincidence of morbid phenomena are, in our opinion, neither sufficiently numerous nor sufficiently clear; we must continue to observe our patients very attentively, and to interrogate them very carefully upon the prodromes. It is not difficult to discover whether this complication of took place at the same time as the rheumatism; or whether any palpitations preceded or followed the rheumatic pains.

With regard to inflammation of the pericardium, we had two fatal cases, one of which presented a complication of rheumatism, the other of typhoid fever. We can draw no conclusion from these results.

As to endo-carditis, it is a very rare disease, whatever may be said of it. We have had some cases of it, within the last few years; we observed a well-marked case of it within the last year; another presented itself in the practice of M. Grisolle whilst he was at the head of our clinic. A third case has been cited by M. Guenau de Mussy; the pathological specimens of the last case, which afforded a great deal of interest, have been deposited in the Dupuytren Museum.

The case cited by M. Grisolles, was that of a woman of a good constitution, although slightly inclined to a lymphatic temperament; she entered the hospital with all the symptoms of an internal phlegmasia of the heart; she was treated in large and frequent bleedings; but, notwithstanding these energetic means, she succumbed. The observations in M. Guenau de Mussy's case referred to a woman of a nervo-sanguineous temperament; she suffered from very acute deep seated pains in the region of the heart; she had never had rheumatism. We employed a very energetic treatment, but were unable to save her. The patient whose case we met with last year, and who had endo-carditis, had not suffered from rheumatism. We are obliged therefore to admit that endo-carditis is not always, nor as often as it is said to be, the result of acute rheumatism, and that it can take place without being complicated with the latter affection. We have already expressed our opinion upon the organic affections of the heart, which are considered as consequent upon rheumatism. This question, in our opinion, is far from being satisfactorily decided. In reference to this matter the following is the result of our experience as to the proportion and relation of these affections.

In five cases rheumatism preceded, twice it followed, one case was doubtful. In the thirty-three cases remaining, of which there were eleven of simple hypertrophy and eleven of lesions of the valves, there was not one case of rheumatism. In fine, in one hundred and thirty-four cases of organic diseases of the heart, which we have observed in these last few years, and carefully collected together, we ascertained in eighteen cases the complication of articular rheumatism, but in these eighteen cases we include that of an idiotic woman, on whose answers we cannot rely with any certainty. This is, as it must appear, so small a proportion, that the cases presenting this complication, may be considered as exceptions. Therefore, even admitting that the heart, as a muscular organ, may be subject to rheumatism, we are led inevitably to the conclusion, according to the actual state of the science, that the coincidence of organic lesions of this organ with articular rheumatism of the limbs is pretty rare, and much more so than it is generally said to be.

### SYPHYLITIC AFFECTIONS.

The number of syphilitic affections which we have had to treat this year has been very small, being accounted for by the fact, that this hospital is not destined for the treatment of these diseases; we have only had some few cases which could serve for clinical instruction. We have been induced to treat of these diseases in this course on account of the great disagreement which has existed among practitioners for some years past, in the management of these affections. It may appear singular perhaps that a physician, not attached to a hospital especially destined to the treatment of those diseases, and who is not consequently as competent to treat them as those physicians who make it their special study, that he should venture to engage himself in such a contest; but as those that are competent differ in opinion among themselves, the examination of the question falls under the general domain of medicine, aud hence it is allowable to every physician charged with clinical instruction, to discuss it. It is even incumbent upon them to avow frankly their opinion, guided by the experience which they have acquired in the matter. Physicians who devote themselves specially and exclusively to the treatment of syphilitic diseases, have undoubtedly a great advantage over others in a practical point of view; but they are not protected from certain influences and causes of error, to which other physicians are much less exposed. Thus, many patients whom they treat, and who, after having been cured to all appearances of certain syphilitic symptoms, leave the hospital, are never more heard of by their physicians. But if these patients have a recurrence of the same affection, they take good care not to return to the same physicians, but they enter, generally, another hospital, or put themselves under another physician. Hence the impossibility for the first physician to obtain any definite results from his treatment, and hence there often ensues a false conviction on the part of the practitioner of the efficacy of his treatment. The primary symptoms, so simple in appearance, last often for a long time; and in order to judge fairly of the efficacy of a particular treatment, we must persevere in its use for a very long time, and continue with it even after all symptoms have disappeared.

Physicians agree pretty generally concerning the primary symptoms; but the same cannot be said in regard to the secondary. It has been said by some that there is no constancy in their appearance, that sometimes they develope themselves, sometimes they do not, without their being able to ascertain the presence of any of those conditions, which can cause this result to vary thus; so that finally, they are forced to delay the treatment of them until they develope themselves. It happens sometimes that these symptoms present themselves, even after a mercurial treatment; we must not conclude from this that mercury is not efficacious in this disease, but that the treatment has not been judiciously managed, or not continued for a sufficient length of time. I speak from experience; I have seen many patients treated, and have treated myself, many who have been affected with syphilis, and I have never seen the morbid symptoms return when they have been subjected to a methodical treatment. I was in the habit of administering anti-syphilitic preparations in small doses long continued. Dupuytren continued his treatment of the secondary symptoms only for the length of time which they took to develope themselves. This rule does not appear to us to be a sufficiently safe one, for if by chance the primary symptoms were to disappear very quickly, and that the secondary symptoms developed themselves in a short time, it would follow that the treatment would have to be a very short one, which in a great number of cases, would afford a sufficient safeguard against a return. Inconvenience results, on the other hand, from giving mercurial preparations in large doses, which is the practice of some physicians; in the first place because the mercury produces gastro-intestinal irritations, more or less severe; secondly, because in large doses it is apt to produce salivation in a very short time; which is a very annoying result, not only because it incommodes the patients, and prevents their sleeping, but also because it interrupts the treatment. During this interruption the syphilitic virus regains all its primitive strength, new symptoms may develope themselves, and we are finally obliged to recommence our treatment as if nothing had been done. The system, therefore, of giving mercury in large doses is a bad one. In giving it, on the other hand, in small doses, we may continue its use without interruption and without any inconvenience for a very long time. We are in the habit of continuing this treatment for five or six months, in giving mercury in the form of the deuto-chloride, in pills, in the dose of one-tenth of a grain night and morning, and the symptoms disappear without our having any apprehension of their return. We cannot insist too much upon the necessity of employing judiciously a similar treatment, and not to delay its application until more serious symptoms develope themselves; for in delaying a treatment, which employed in the right time may be very simple and very efficacious, we expose ourselves at a later period to see circumstances arise which will render it much more difficult, and often even useless. Thus, for example, how many individuals do we see, who having subjected themselves to no judicious treatment during their youth, delay taking care of themselves until they are on the eve of being married. To what serious inconveniences or painful preoccupations do these persons then find themselves subject.

As to the treatment of the secondary symptoms, we employ the mercurial preparations in the cases of those individuals, who have not as yet undergone any treatment, whilst in the case of those who have already been treated by these preparations, we prefer employing the iodide of potassium, which has afforded the greatest success in our hands, as well as in those of many other practitioners. Employed in a judicious manner, we have nearly always seen it in the end triumph over the secondary and tertiary symptoms, even those of the most acute and chronic kind; we have seen it recently relieve the pains in the bones, overcome wakefulness, and thus afford the greatest relief to the afflicted, even in those cases where it may not be entirely efficacious.

### TUBERCULAR AFFECTIONS.

We have had sixty-seven cases of tubercular affections of the lungs, nearly the half of which proved fatal. Forty-seven cases presented themselves in the six months of summer, and twenty in those of winter; eighteen cases were among the men, and forty-seven among the women. Notwithstanding the disproportion of the number of beds in the two wards, the number of women afflicted with tubercular affections, as is seen, is in a much greater proportion. It is, however, about the usual proportion.

About one half of these patients had not passed the age of thirty five, three were more than sixty years old, which proves that this disease, though much less liable to attack those advanced in years, is however sometimes met with in these persons, and that no age is absolutely exempt from it. Among those cases terminating fatally, there was one attacked with a most dreadful hæmoptysis; it very seldom happens that phthisical patients, though subject to these kinds of hemorrhages, succu nb immediately from the effects of this symptom. There have been two cases of pneumothorax; this is a very serious complication and generally fatal; the cause of its fatality is easily understood.

#### *Treatment.*

We shall not go into the details of the treatment in reference to certain symptoms of phthisis, such as sweats, the cough, the diarrhœa, etc., for which we must employ different means according to the particular indications. We shall confine ourselves to some considerations upon the general treatment. The causes of tubercles are as yet sunk in the deepest obscurity; and we are forced to give to this affection, for want of well determined causes, a spontaneous origin. The influence of inheritance upon the development of this disease is well ascertained. It is well known that the children of a phthisical mother are generally most subject to the same affection. But to what point does this influence exercise itself? This is a difficult problem to solve. It is also known that certain climates exercise a serious influence over this disease; but we know not at the same time, to what degree, and in what respect these climates are unfavourable. Experiments have been made upon animals, in order to develope tubercles artificially: they have been successful to a certain degree; but the results of these experiments are not in our opinion, applicable to the human species, so that we can draw no positive conclusion in regard to the question before us.

It is well known also that inflammation of the lung, that of the pleura, the eruptive diseases and other similar affections, favour very much the development of tubercles, and hasten their progress and their fatal termination; hence the precise indication is to combat these serious complications, and to place the tubercles in the most favourable physiological conditions, at least whenever it is possible; for we cannot combat efficaciously these phlegmasiæ without the aid of antiphlogistic means, and often we have to deal with individuals already very much enfeebled, and who cannot bear bloodletting. The chief point of attack should be directed against the tuberculous affection itself; this is undoubtedly very difficult, but not absolutely impossible.

Phthisis is not, in our opinion, entirely beyond all the resources of art and nature, as some other diseases, cancer for instance. We think that it is not absolutely incurable, and that at some period the means will be found to combat it efficaciously. There are tubercular affections which are cured with comparative ease. Those scrofulous tumours found in children are cured daily; and what happens in the tubercular ganglions, may very well take place in the other organs.

Is the tubercular matter susceptible of resolution? Can it, after having been naturally deposited in the tissues, be retaken by means of the absorbents in the economy? As respects these questions, what is certain and proved by experience, is that the pulmonary tubercles may become softened, that the sac where this morbid matter is collected may empty itself, contract, and throw out a species of false membrane of cicatrization.

It is also ascertained that finally the softened tubercles are transformed into cretaceous masses which become inoffensive, and whose presence is not at all detrimental to the preservation of more or less perfect health. Proof of these facts exist; we can merely explain them by hypothesis. Cases of tuberculous lungs, have been seen in autopsies, and indeed often cited in presenting empty cavities and cicatrices in subjects in which had been detected during life and long before death, the cavernous râle and the gurgling sound characteristic of tubercular softening. Pulmonary tubercles are therefore susceptible of cure. This is a point upon which there exists no doubt. Why then is the disease so seldom cured? Because that cause which produces one tubercle, will produce a thousand, if permitted to act, and unfortunately the means of paralyzing its action have been hitherto but very slightly efficacious, because these tubercles develope themselves in several points at the same tim) in consequence of the existence of a particular tendency to tuberculization in the organism, and the functional and organic disorders which result from it are so great that death is inevitable.

The chief point in the treatment of this affection is then to remove all causes tending to produce or develope the fatal germ. To attain this every thing has been tried; all the materia medica has been called into requisition. The multitude of remedies proposed in the treatment of this disease, proves sufficiently by itself what the nature of this affection is, and how difficult is its cure. The preparations of sulphur, the preparations of iodine have been sometimes employed with some success; but in the cases of cure which have been cited, is it to the remedies employed that we must attribute the success? is it not rather to the resources of the vis medicatrix naturæ? Setons, counter-irritants, fumigation of balsamic substances, as also the internal employment of certain substances, have sometimes been advantageous; they have procured some relief. But the good effects derived from them are not sufficiently constant or multiplied for us to decide positively in their favour. In short, all the remedies furnished by the materia medica have hitherto generally failed. It was necessary, consequently, to employ other means; recourse has been had principally to hygiene.

Mineral waters have been employed, principally those which abound in the salts of sulphur; thus the waters of Bonnes have been used and extolled, as also the waters of Bareges, Luchou, &c.; finally, lately the waters of Chelle (in Savoy) have been very much extolled, which appear to be superior to the others in consequence of a combination of sulphur and iron which they contain. All these waters appear to exercise a happy influence upon the phlegmasia when it exists in the first degree. In fine, wherever a remedy is not dangerous, we must not neglect it; we must try it, above all, when we are treating a disease which resists all known remedies.

Hygiene produces in these cases effects, sometimes, which the materia medica does not; we place a great deal of confidence in hygienic measures well directed, in the change of climate, for example, in voyages. This is also the opinion of all experienced practitioners. But what is the influence of a new climate? Is it owing entirely to a change of air? It is probable that the air is but one of the elements in this favourable condition of change of climate. In fact, when we change climate, we do not only change air, but every thing, food, habits, manner of living, in a word, we find ourselves in a new sphere of action, of ideas, and of impressions which must necessarily act on the organism and modify it very much. Thus have we sometimes seen poor unfortunate sufferers embarking, in an almost dying state, for a long voyage, find themselves immediately much better, breathe with more ease in the midst of the ocean, and then return after a distant voyage, if not entirely cured, at least in an infinitely better condition than before their departure. How do these causes act? We are as ignorant of their action as of the development of the tubercle, which under the influence of some causes manifest themselves in the organism in the flower of youth. It is but natural to suppose that a change of climate, a new mode of living, a complete change in all the hygienic measures, are all new conditions, which must necessarily produce great modifications in the economy; that these are also capable of modifying the natural tendency in certain persons to the production of tubercles, or to arrest their evolution. It is therefore a rational practice, to advise, as much as the particular circumstances of the case permit it, a change of climate and distant voyages. Experience authorizes us to anticipate the greatest advantages from the practice.

*Paris, August,* 1843.

